# Museos precarios: memorias, olvidos y aprendizajes en la Patagonia argentina

**DOI:** 10.1590/S0104-59702024000100032

**Published:** 2024-07-15

**Authors:** Emiliano Nicolás Abad García

**Affiliations:** i Departamento de Historia Contemporánea/Universidad Autónoma de Madrid. Madrid – Spain emiliano.abad@predoc.uam.es

El libro constituye un ejercicio crítico, plural y reflexivo sobre el proceso de construcción de museos locales y comunitarios en la Patagonia argentina, con especial atención a la provincia de La Pampa. En total, se trabajan más de veinte museos, entre los que se encuentran el Museo Regional Pampeano, el Museo El Castillo de Parque Luro y el Museo Histórico Municipal de Alpachiri. Las preguntas emergen como una constante, rayando el imperativo: ¿cuándo y cómo nacieron estos museos? ¿A qué desafíos y dificultades tuvieron que hacer frente? ¿Qué historias cuentan y, sobre todo, para quiénes las cuentan? Otras preguntas siguen la estela de los procesos, pero se centran más en los protagonistas: ¿cuál ha sido y cuál sigue siendo el rol de los profesionales – historiadores, arqueólogos, museólogos etc. – en la construcción de estos museos? ¿Y los del Estado y sus intermediarios, especialmente a nivel local y comunitario? Por último, ¿cómo intervienen o han intervenido todos los saberes *amateurs* de los aficionados, sin cancelar los saberes no hegemónicos de otros agentes, como los herederos de las poblaciones indígenas exterminadas durante la Conquista del Desierto (1878-1885)? ¿Cómo se ha construido un diálogo – si es que dicho diálogo ha existido – entre los principales responsables de cada museo y el resto de vecinos, muchos de ellos atravesados por relaciones de poder basadas en discursos sobre la raza, el origen o la nación, relaciones que todavía configuran nuestra experiencia contemporánea de la vida en comunidad?

El libro se divide en dos partes. La primera, de ocho capítulos, se centra en el desarrollo de un gran *corpus* teórico sobre museos y su evolución historiográfica, para luego adentrarse en el estudio de casos concretos, como el Museo de la Patagonia, la Casa Vicente de Miguel Riglos o el Museo del Pueblo de Toay. La segunda, de cuatro capítulos, está dedicada a la exposición de relatos en primera persona de grandes hacedores de proyectos locales, como Daniel Pincén – director del Museo Provincial de Historia Natural **–** o Claudia Patricia Visbeek, gestora, técnica y miembro del equipo fundacional del Museo de la Comunidad de Winifreda. Si la primera parte ya es novedosa, sobre todo si se quiere comparar la Patagonia con otros países o regiones, la segunda constituye un gesto mucho más plural y democrático, al darle voz a los protagonistas. En el libro participan 13 autores, de los cuales solo dos son hombres. Más allá del reduccionismo – subjetivo y de género –, no podemos dejar de insistir que el trabajo en museos y su estudio a través de enfoques como los *museum studies* está cada vez más marcado por el esfuerzo intelectual femenino. Como bien señala la autora, María Silvia Di Liscia (2022), este es un fenómeno que ya ha sido analizado en otros estudios, pero con la particularidad de que ahora se reproduce al interior del libro. Los museos, especialmente a nivel local y comunitario, siguen siendo un nicho de género, como la atención social o la enseñanza en docencia, todos ámbitos relativamente poco remunerados y, salvo excepciones – como los puestos de dirección o con mayor reconocimiento a nivel personal y simbólico –, poco codiciados por los varones. Al igual que la educación o la economía de los cuidados, los museos comunitarios son lugares paradójicos, al ser espacios marginales en la consideración social y, sin embargo, de vital importancia para la reproducción de la memoria, los valores y las aspiraciones de una comunidad. Después de todo, ¿quién no ha visitado alguna vez un museo, siendo apenas un niño o un proyecto de ciudadano?

Fragilidades. La gran mayoría de museos trabajados en este volumen son museos precarios, muchos casi sin personal, sin estructura y sin presupuesto. Son museos pequeños, pero que, contra todo pronóstico, transmiten una fuerza – y, en muchos casos, una presencia invisible – que ya quisieran tener muchos de los grandes proyectos de las principales capitales del mundo. Su fuerza reside en la inercia de un entramado social que, con sus propios tiempos, con sus propios anhelos y limitaciones, persiste en una larga memoria comunitaria no exenta de dolor y miseria. El problema, y esta parece ser la tesis central del libro, es que, en un contexto supuestamente plural y democrático, casi todos estos museos continúan legitimando relatos en donde los primeros pobladores – *blancos* y de origen casi siempre europeo – encarnan un pasado en donde todo podía lograrse con mucho esfuerzo y dedicación, pero en donde jamás se discute y a veces directamente se ocultan los eventos más traumáticos y conflictivos de su propia historia. ¿Y cuáles son estos eventos? Tres, como mínimo: la conquista y la última dictadura – las dos protagonizadas por los grandes héroes del gran relato mitológico nacional: el Ejército –, sin olvidarnos de la fiebre neoliberal de la década de 1990, que despojó a muchos de estos pueblos de uno de los máximos símbolos del “progreso y la civilización”: el ferrocarril.

Perdidos y asilados. El libro desplaza su mirada hacia los silencios ocultos en los museos y en cómo estos silencios están enraizados en la vida comunitaria. Sin conquista, sin ferrocarriles y sin militares ninguno de estos pueblos se hubiera jamás construido (o no tal y como los conocemos). La ficción es irrefutable y, precisamente por ello, nos lleva a preguntarnos por qué la sociedad en su conjunto no puede, no logra y/o no desea resignificar eventos como la conquista o la dictadura. La alternativa, sugerida en varios pasajes del libro, es la construcción de una relación nostálgica con el pasado – que nunca fue ni tan próspero ni tan esplendoroso –, hasta convertirlo en una especie de tesoro impenetrable que poco contribuye al desarrollo de una ciudadanía crítica o mínimamente responsable. ¿Por qué? Porque no reconoce el conflicto ni la diferencia, dos componentes centrales de toda lógica democrática. Como ciudadano e historiador, estoy convencido de que hay que exponer, deconstruir y desnaturalizar estos relatos, porque nunca hay única verdad, sino que la verdad también se juega en la relación con los otros y con nuestro propio pasado. Por eso este libro es tan importante. Tan oportuno. Tan necesario.
